# A Baseline for Cross-Database 3D Human Pose Estimation

**DOI:** 10.3390/s21113769

**Published:** 2021-05-28

**Authors:** Michał Rapczyński, Philipp Werner, Sebastian Handrich, Ayoub Al-Hamadi

**Affiliations:** Neuro-Information Technology Group, Otto von Guericke University, 39106 Magdeburg, Germany; Philipp.Werner@ovgu.de (P.W.); Sebastian.Handrich@ovgu.de (S.H.); Ayoub.Al-Hamadi@ovgu.de (A.A.-H.)

**Keywords:** 3D human pose estimation, deep learning, generalization

## Abstract

Vision-based 3D human pose estimation approaches are typically evaluated on datasets that are limited in diversity regarding many factors, e.g., subjects, poses, cameras, and lighting. However, for real-life applications, it would be desirable to create systems that work under arbitrary conditions (“in-the-wild”). To advance towards this goal, we investigated the commonly used datasets HumanEva-I, Human3.6M, and Panoptic Studio, discussed their biases (that is, their limitations in diversity), and illustrated them in cross-database experiments (for which we used a surrogate for roughly estimating in-the-wild performance). For this purpose, we first harmonized the differing skeleton joint definitions of the datasets, reducing the biases and systematic test errors in cross-database experiments. We further proposed a scale normalization method that significantly improved generalization across camera viewpoints, subjects, and datasets. In additional experiments, we investigated the effect of using more or less cameras, training with multiple datasets, applying a proposed anatomy-based pose validation step, and using OpenPose as the basis for the 3D pose estimation. The experimental results showed the usefulness of the joint harmonization, of the scale normalization, and of augmenting virtual cameras to significantly improve cross-database and in-database generalization. At the same time, the experiments showed that there were dataset biases that could not be compensated and call for new datasets covering more diversity. We discussed our results and promising directions for future work.

## 1. Introduction

Three-dimensional human body pose estimation is useful for recognizing actions and gestures [1,2,3,4,5,6,7,8], as well as for analyzing human behavior and interaction beyond this [9]. Truly accurate 3D pose estimation requires multiple cameras [10,11,12], special depth-sensing cameras [13,14,15], or other active sensors [16,17,18], because with a regular camera, the distance to an object cannot be measured without knowing the object’s actual scale. However, many recent works have shown that 2D images suffice to estimate the 3D pose in a local coordinate system of the body (e.g., with its origin in the human hip). Applications such as the recognition of many actions and gestures do not require the accurate position of the body in the 3D world, so local (also called relative) 3D pose estimation from 2D images can be very useful for them.

Due to the challenges of obtaining accurate 3D ground truths, most prior works used one or two of the few publicly available databases for 2D-image-based 3D pose estimation, such as: Human3.6M [19,20], HumanEva-I and HumanEva-II [21,22], Panoptic Studio [10,11], MPI-INF-3DHP [23], or JTA [24]. All these databases were either recorded in a laboratory (a few sequences of MPI-INF-3DHP were recorded outdoors, but the diversity is still very limited) or synthesized and do not cover the full diversity of possible poses, peoples’ appearances, camera characteristics, illuminations, backgrounds, occlusions, etc. However, for real-life applications, it would be desirable to create 3D pose estimation systems that work under arbitrary conditions (“in-the-wild”) and are not tuned to the characteristics of a particular limited dataset. Reaching this goal requires much effort, probably including the creation of new datasets. However, one step towards better in-the-wild performance is discussing dataset biases and measuring cross-database performance, that is training with one database and testing with another one [25]. This step was addressed in our paper.

Our key contributions are as follows:

1. We reviewed the literature (Section 2) and discussed biases in the commonly used datasets Human3.6M, HumanEva-I, and Panoptic Studio (Section 3), which we also used for our cross-dataset experiments;
2. We proposed a method for harmonizing the dataset-specific skeleton joint definitions (see Section 4.1). It facilitates cross-dataset experiments and training with multiple datasets while avoiding systematic errors. The source code is available at https://github.com/mihau2/Cross-Data-Pose-Estimation (accessed on 27 May 2021);
3. We proposed a scale normalization method that significantly improves generalization across cameras, subjects, and databases by up to 50% (see Section 4.2). Although normalization is a well-known concept, it has not been consistently used in 3D human pose estimation, especially with the 3D skeletons;
4. We conducted cross-dataset experiments using the method of Martinez et al. [26] (Section 5), showing the negative effect of dataset biases on generalization and the positive impact of the proposed scale normalization. Additional experiments investigated the effect of using more or less cameras (including virtual cameras), training with multiple datasets, applying a proposed anatomy-based pose validation step, and using OpenPose as the basis for the 3D pose estimation. Finally, we discussed our findings, the limitations of our work, and future directions (Section 6).

## 2. Related Work

Since the work of Shotton et al. [13] and the development of the Kinect sensor, enormous research efforts have been made in the field of human pose estimation. While the work at that time was often based on depth sensors, approaches developed in recent years have focused primarily on estimating the human pose from RGB images. In addition to the high availability of the corresponding sensors, which allows for the generation of extensive datasets in the first place, this is primarily due to the development in the area of deep neural networks, which are very successful in processing visual information. Therefore, all current approaches are based on deep neural networks, but, according to their objectives, can be roughly divided into three categories.

The quantitative results of prior works are summarized in Table 1, Table 2 and Table 3 for reference.

### 2.1. 2D Human Pose Estimation

The first class of approaches aims to predict the 2D skeleton joint positions from an RGB input image. In their approach called convolutional pose machines [27], the authors proposed a network architecture of cascading convolutional networks to predict belief maps encoding the 2D joint positions, where each stage refines the prediction of the previous stage. This approach was extended by Newell et al. [28] by replacing the basic convolutional networks with repeated bottom-up, top-down processing networks with intermediate supervision (stacked hourglass) to better consolidate features across all scales and preserve spatial information at multiple resolutions. In [29], the pose estimation problem was split into two stages. A base network with a pyramidal structure aimed to detect the 2D joint positions, while a refinement network explicitly learned to predict the “hard-to-detect” keypoints, i.e., keypoints that were not detected by the base network during the training process. In addition to 2D keypoints, the network in the part affinity field approach [30] learns to predict the orientation and location of several body parts (limbs), resulting in superior keypoint detection. This is particularly helpful when it comes to associating multiple detected joint positions with individuals in multi-person scenarios. This approach was later integrated into the OpenPose framework [31]. In [32], the authors replaced the discrete pixelwise heat map matching with a fully differentiable spatial regression loss. This led to an improved pose estimation, as the low resolution of the predicted heat maps no longer limited the spatial precision of the detected keypoints. Furthermore, several regularization strategies increasing the prediction accuracy were proposed. Human pose estimation in multi-person scenarios poses a particular challenge. Top-down approaches (e.g., [33]) perform a person detection (bounding boxes) followed by a single-person pose estimation, but typically suffer from partial or even complete overlaps. In contrast, bottom-up approaches [34] first detect all joint positions and then attempt to partition them into corresponding person instances. However, this requires solving an NP-hard partitioning problem. The authors in [35] addressed this problem by simultaneously modeling person detection and joint partitioning as a regression process. For this purpose, the centroid of the associated person was predicted for each pixel of the input image. In [36], the authors first identified similarities among the several approaches for human pose estimation and provided a list of best practices. In their own approach, the authors achieved a state-of-the-art performance by replacing upsample layers with deconvolutional filters and adding optical flow for tracking across multiple images. Whereas all other approaches obtain high-resolution representations by recovering from low-resolution maps using some kind of upscaling networks, Sun et al. [37] proposed HRNet, a network architecture that is able to maintain high-resolution representations throughout all processing steps, leading to superior performance on 2D human pose estimation.

### 2.2. 3D Human Pose Estimation from 2D Images

The next class of approaches predicts 3D skeleton joint positions using raw 2D RGB images as the input. Li and Chan [69] used a multitask learning approach to simultaneously train a body part detector and a pose regressor using a fully connected network. In contrast to the direct regression of a pose vector, Pavlakos et al. [68] transferred the idea of the heat map-based 2D pose estimation into the 3D domain and predicted per-joint 3D heat maps using a coarse-to-fine stacked hourglass network, where each voxel contains the probability that the joint is located at this position. Each refinement stage increases the resolution of the z-prediction. Tekin et al. [52] proposed fusing features extracted from the raw image with features extracted from 2D heat maps to obtain a 3D pose regression vector. A similar approach was developed in [40], but instead of deriving features from an already predicted heat map, the authors utilized latent features from the 2D pose regression network. Their end-to-end trainable approach allows for sharing common representations between the 2D and the 3D pose estimation tasks, leading to an improved accuracy. Dabral et al. [51] utilized the same architecture as in [40], but introduced anatomically inspired loss functions, which penalize pose predictions with illegal joint angles and non-symmetric limb lengths. In LCR-Net [46], the pose estimation problem was split into three subtasks: localization, classification, and regression. During localization, candidate boxes and a list of pose proposals are generated using a region proposal network. The proposals are then scored by a classifier and subsequently refined by regressing a set of per-anchor-pose vectors. The subnets share layers so that the complete process can be trained end-to-end. Kanazawa et al. [70] took a slightly different approach. Instead of keypoints, the authors aimed to predict a full 3D mesh by minimizing the reconstruction error. Since the reconstruction loss is highly underconstrained, the authors proposed an adversary training to learn whether a predicted shape is realistic or not. Sun et al. [42] evaluated the performance of the differentiable soft-argmax operation as an alternative to the discrete heat map loss in greater detail and verified its effectiveness. Their approach achieved state-of-the-art results on Human3.6M by splitting the volumetric heat maps into separate x-, y- and z-maps, which allowed for mixed training from both 2D and 3D datasets. Instead of directly dealing with joint coordinates, Luo et al. [44] modeled limb orientations to represent 3D poses. The advantage is that orientations are scale invariant and less dependent on the dataset. Their approach achieved good results on several datasets and generalized well to unseen data. In [49], the authors combined action recognition with human pose estimation. The proposed multitask architecture predicted both local appearance features, as well as keypoint positions, which were then fused to obtain the final action label. The actual pose estimation was based on heat maps and the soft-argmax function. The approach showed state-of-the art results on both pose estimation and action recognition. Another multitask approach was presented by Trumble et al. [71]. It simultaneously estimates 3D human pose and body shape using a symmetric convolutional autoencoder. However, the approach relies on multi-view camera inputs. Approaches that adapt a kinematic skeleton model to the input data typically rely on the detection of corresponding points. This task has been mostly addressed in scenarios where a depth sensor was available. In contrast to this, DensePose [72] maps an input image to the 3D surface of the human body by regressing body part-specific UV coordinates from each RGB input pixel. The approach showed good results, but one has to keep in mind that identifying correspondences is not yet a complete pose estimation due to possible 2D/3D ambiguities and model constraints. All aforementioned approaches learned a direct mapping between the input data and the pose to be estimated. This must be distinguished from approaches that initially learn a latent representation of either the input or the output data [56,73]. In [56], an overcomplete autoencoder network was used to learn a high-dimensional latent pose representation. The input image was then mapped to the latent space, leading to a better modeling of the dependencies among the human joints. In contrast, Rhodin et al. [73] trained a latent representation of the input data by utilizing an autoencoder structure to map one camera view to another. The pose was then regressed from the latent state space. The approaches showed good, but not the best results. Sárándi et al. [60] demonstrated the effectiveness of data augmentation. By occluding random positions in the RGB image with samples from the Pascal VOC dataset, the mean per-joint position error (MPJPE) can be reduced by up to 20%, making this approach the ECCV pose estimation challenge winner in 2018. The occlusion acts as a regularizer, forcing the network to learn joint positions from several visual cues. The authors used ResNet as the backbone architecture to generate volumetric heat maps. As high-resolution volumetric heat maps are quite memory intensive, the authors of MargiPose [47] proposed to learn three distinct heat maps instead. The maps represent the xy-, xz-, and yz-plane and can be seen as projections of the volumetric heat map. Their approach, which was based on the Inception v4 model, achieved good results and provided a memory-efficient alternative to volumetric heat maps. Habibie et al. [38] contributed by integrating 3D features in the latent space of the learning process. The regressed 3D pose is back-projected to 2D before the loss is computed and thus allows a 3D pose estimation based on 2D datasets. However, there is no explicit supervision of the hidden feature maps that encode the 3D pose cues. A recent work by Wu and Xiao [59] proposed to model the limbs explicitly. Their approach was somewhat similar to OpenPose [31], but extended it to the 3D domain. Next to 2D keypoints from 2D heat maps, the network learned to predict densely-generated limb depth maps. Latent features from the 2D pose estimator and the depth map estimation, as well as 3D specific additional features were then fused to lift the 2D pose to 3D. Their approach significantly outperformed all other methods on the Human3.6M and MPI-INF-3DHP datasets.

### 2.3. 3D Human Pose Estimation from the 2D Pose

The last class of approaches attempts to predict the 3D pose from an earlier predicted 2D pose, a process typically known as lifting. A big advantage of separating the lifting from the 2D pose estimation is that it can be pre-trained using synthetic poses. Martinez et al. [26] directly regressed 3D poses from 2D poses using only fully connected layers. Their approach achieved excellent results, at least when using 2D ground truth joint positions as the input. In [41], the authors built a huge library of 3D poses and matched it against a detected 2D pose. Using also the stored camera parameters, the best 3D pose was then scaled in a way that it matched the 2D pose. Pavllo et al. [74] exploited temporal information by using dilated temporal convolutions on 2D keypoint sequences. Hossain and Little [75] designed an efficient sequence-to-sequence network taking a sequence of 2D keypoints as the input to predict temporally consistent 3D poses. Their approach achieved state-of-the-art results for every action class of the Human3.6M dataset. While CNNs are suitable for processing grid-like input data (e.g., images), graph convolutional networks (GCNs) can be seen as a generalization of CNNs acting on graphs. In [76], Zhao et al. exploited the hierarchical structure of skeletons by describing both 2D and 3D skeletons as graphs and used CGNs to obtain 3D poses from 2D poses. The aforementioned approaches reported excellent results, in particular when temporal information was used. However, they heavily relied on the quality of the underlying 2D pose estimator. If no 2D ground truth was used, the accuracy was typically similar to approaches that obtained 2D and 3D poses directly from the image.

### 2.4. Cross-Dataset Generalization

Comprehensive datasets are required in order to train methods for pose estimation. In contrast to 2D pose estimation, reliable 3D pose data cannot be obtained by manually annotating images taken in-the-wild, but are acquired with the help of motion capture systems (e.g., VICON [77], The Captury [78], IMU). This typically limits the acquisition to controlled in-the-lab environments with low variations in terms of subjects, camera view points, backgrounds, occlusions, lighting conditions, etc. This raises the questions how well these approaches (a) perform across multiple controlled datasets and (b) generalize to unconstrained in-the-wild data. Work in this area is still limited. The typical approach is to combine in-the-wild 2D pose data with in-the-lab 3D pose data. Mehta et al. [23] used transfer learning to transfer knowledge from a pre-trained 2D pose network to a 3D pose regression network [23]. They further provided the MPI-INF-3DHP dataset, an augmented in-the-wild 3D pose dataset, by utilizing a marker-less multi-camera system [78] and chroma keying (green screen). The best results on Human3.6M were achieved using transfer learning and including additional data from MPI-INF-3DHP. Zhou et al. [40] mixed 2D and 3D data per batch to learn common representations between 2D and 3D data by computing additional depth regression and anatomical losses for 3D training samples [40]. When additional 2D pose data from the MPII dataset were included, errors on the Human3.6M dataset were reduced by up to 15 mm, and the proportion of correctly estimated joints (PCKs) increased from 37.7% to 69.2% on the MPI-INF-3DHP dataset. This indicated that the constrained setting of Human3.6M is insufficient to generalize to in-the-wild data. The authors also concluded that adding additional 2D data did not improve the accuracy of the 2D pose prediction, but mostly benefited the depth regression via shared feature representations. As mentioned above, Habibie et al. [38] circumvented the problem of missing 3D pose labels by learning both view parameters and 3D poses. The 3D poses were then back-projected to 2D (using a trainable network) before applying the 2D loss. Their approach showed high accuracy and generalized well to in-the-wild scenes. Other approaches attempt to generate 3D labels from existing 2D datasets. Wang et al. [79] achieved this by first mapping a 2D pose to 3D using a “stereo-inspired” neural network and then refined the 3D pose using a geometric searching scheme so that the determined 3D pose matched the 2D pose with pixel accuracy. In [80], which was an updated version of [46], Rogez et al. [46] created pseudo 3D labels for 2D datasets by looking for the 3D pose that best matched a given 2D pose in a large-scale 3D pose database. Further work addressed the problem of missing 3D pose labels by generating synthetic datasets by animating 3D models [81,82] or rendering textured body scans [83]. While rendering may seem promising, both integrating human models in existing images, as well as rendering realistic scenes are not trivial and often require a domain adaption to generalize from synthetic to real images [81,83,84]. Therefore, Rogez and Schmid [85] proposed to build mosaic pictures of real images from 2D pose datasets. While artificial looking, the authors showed that CNNs can be trained on these image and generalize well to real data without the need for any fine-tuning and domain adaption.

While many authors combined multiple training datasets, work on cross-dataset evaluation is still limited. To the best of our knowledge, the very recent work of Wang et al. [86] was the first to systematically examine the differences among existing pose datasets and their effect on cross-database evaluation. However, they focused on systematic differences of camera viewpoints and conducted their experiment with another set of databases, compared to our work.

### 2.5. Non-Vision-Based Approaches

All approaches listed so far were based on optical sensors, i.e., cameras. We would like to point out to the reader that besides visual methods, other ranges of the electromagnetic spectrum can also be used to estimate the human pose. The major advantage of these approaches is that they are independent of lighting, background, as well as clothing and even allow for person detection and pose estimation through walls and foreground objects. Moreover, privacy issues can be avoided in contrast to camera-based approaches. The most prominent examples are microwaves and millimeter waves. In [16], the authors proposed a radar-based approach (operating in the 5.56–7.25 GHz range) for indoor person location, obtaining a spatial resolution of 8.8 cm. In RFPose [17], the authors utilized radio frequency (RF) signals (20 kHz–300 GHz) and visual information to extract 2D skeleton positions. The approach was later extended to 3D [87], where the authors reported a mean per-joint localization error of 4.2 cm, 4.0 cm, and 4.9 cm for the X-, Y-, and Z-axes, respectively. However, a major disadvantage of this approach is the very specific and high hardware requirements (synchronized 16 + 4 T-shaped antenna array with frequency-modulated continuous waves), which severely limit its possible applications. There are also LIDAR-based approaches (e.g., [18]), but these are usually expensive and power consuming. More recently, WiFi-based approaches were proposed. In [88], Wang et al. [88] developed a human pose estimation system, which reconstructed 2D skeletons from WiFi by mapping the WiFi data to 2D joint heat maps, part affinity fields, and person segmentation masks. The authors reported an average percentage of correctly detected keypoints (PCK) of 78.75% (89.48% for OpenPose [31]). However, their approach performed significantly worse in untrained environments (mPCK = 31.06%). This is a main challenge for all WiFi-based approaches, as WiFi signals exhibit significantly different propagation patterns in different environments. To address this issue and achieve cross-environment generalization, the authors of WiPose [89] proposed to utilize 3D velocity profiles obtained from WiFi signals in order to separate posture-specific features from the static background objects. Their approach achieved an accuracy of up to 2.83 cm (mean per-joint position error), but is currently limited to a single non-moving person.

Camera-based approaches are passive methods, as they capture the ambient light reflected by an object. In contrast, RF-based methods can be considered as active methods, since an illumination signal is actively emitted and interacts with the objects in the scene before being reflected and measured by the receiver. Here, the active illumination signal is often based on appropriately modulated waves or utilizes stochastic patterns. A major drawback of this approach is that the active signal is not necessarily ideal for the specific task, i.e., it is not possible to distinguish between relevant and irrelevant information during the measurement process.

This leads to the idea of *learned sensing* [90], in which the measurement process and the processing of the measurement data are optimized in an overall system. This requires the availability of programmable transmitter hardware whose configuration is determined using machine learning methods in such a way that the emitted illumination signal is optimal for the respective measurement process. This approach has recently been successfully implemented for person recognition, gesture recognition, and human pose estimation tasks. See [91,92] for further details. The idea of *learned sensing* was also applied in the optical domain in order to determine optimal illumination patterns for specific microscopy tasks [93].

For human pose estimation, the learned sensing approach cannot easily be transposed to optical sensors. This is mainly due to the fact that changes in the active illumination signal can be perceived by humans, which is typically undesirable in real-world scenarios. Nevertheless, we suspect that the method can be transferred to approaches that use special (infrared) photodiodes to determine the pose [94]. Furthermore, there may be an application opportunity in multi-camera scenarios. These are often associated with a costly measurement process (high energy consumption, data volume, latency), whereas only a specific part of the measured data is actually required to resolve potentially occurring ambiguities.

## 3. Datasets

In the following subsections, we describe the three 3D human pose estimation datasets that we used in this article: HumanEva-I, Human 3.6M, and Panoptic. Afterwards, we compare the datasets and discuss dataset biases.

### 3.1. HumanEva-I (HE1)

In 2006 and 2010, Sigal et al. [21,22] published the HumanEva-I and HumanEva-II datasets to facilitate the quantitative evaluation and comparison of 3D human pose estimation algorithms. We used HumanEva-I, which is larger and more diverse than HumanEva-II. In HumanEva-I, each of four subjects performs six actions (walking, jogging, gesturing, throwing/catching a ball, boxing, and a sequence of several actions) while being recorded with seven cameras. The ground truth positions of the 15 provided skeleton joints were obtained with a motion capture system using reflective markers.

### 3.2. Human3.6M (H36M)

Ionescu et al. [19,20] collected and published Human3.6M, which is comprised of 3.6 million frames showing diverse body poses of actors performing 15 everyday actions including conversations, eating, greeting, talking on the phone, posing, sitting, smoking, taking photos, waiting, and walking. It total, eleven actors were involved, but they performed individually one after another (i.e., only one person was visible in each video). The data were recorded with four color video cameras and a marker-based motion capture system, providing thirty-two skeleton joint positions.

### 3.3. Panoptic (Pan)

Aiming at analyzing social interaction, Joo et al. [10,11] recorded the Panoptic Studio dataset. In its current state (Version 1.2), it is comprised of 84 sequences with more than 100 subjects. The sequences are very diverse, among others covering: social games (Haggling, Mafia, and Ultimatum) with up to eight subjects; playing instruments; dancing; playing with toddlers; and covering range of motion. In contrast to the other datasets, there is no categorization or segmentation of the actions (beyond the above-mentioned categories of sequences). To record the dataset, Joo and colleagues built the Panoptic Studio, a special dome with more than 500 cameras in its walls. Using these cameras, Joo et al. [10,11] developed an algorithm for obtaining multi-person 3D skeleton joint ground truths without markers. Their algorithm was based on 2D body pose estimation providing “weak” proposals, triangulation and fusion of the proposals, and temporal refinement.

### 3.4. Comparison and Dataset Biases

Computer vision datasets are created for quantitatively measuring and comparing the performance of algorithms. However, “are the datasets measuring the right thing, that is, the expected performance on some real-world task?,” Torralba and Efros asked in their article on dataset biases [25]. We were interested in the task of relative 3D human body pose estimation in the real world, not only in a specific laboratory. Therefore, we may ask if the error in estimating poses on a specific dataset resembles the expected error in real-world application. Are these datasets representative samples of real-world data or are they biased in some way?

Currently, most “in-the-wild” datasets are collected from the Internet, including datasets commonly used for 2D human body pose estimation [95,96]. Although these datasets are very diverse, they may still suffer from biases compared to the real world, e.g., capture bias (pictures/videos are often taken in similar ways) or selection bias (certain types of images are uploaded or selected for datasets more often) [25].

**Figure 1:**
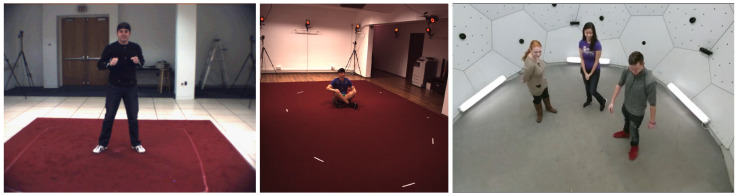
Example images of the HumanEva-I (**left**), the Human3.6M (**middle**), and the Panoptic databases (**right**).

The datasets of 3D pose estimation are less diverse. They are typically recorded in a laboratory, because (1) multi-view camera systems are state-of-the-art for measuring accurate 3D ground truths and (2) building, installing, and calibrating these systems requires much effort (making it hard to move the systems). All three datasets, HumanEva-I, Human3.6M, and Panoptic, were recorded in such an indoor laboratory with very controlled conditions; see Figure 1 for some example images. The datasets differ in size and diversity, as summarized in Table 4. Compared to in-the-wild data, the three datasets suffer from several biases:

- Lighting: The recordings are homogeneously lit, typically without any overexposed or strongly shadowed areas. Further, there is no variation in lighting color and color temperature. Real-world data are often more challenging, e.g., consider an outdoor scene with unilateral sunlight or a nightclub scene with colored and moving lighting;
- Background: The backgrounds are static and homogeneous. Real-world data often include cluttered and changing backgrounds, which may challenge the computer vision algorithms more;
- Occlusion: In real-world data, people are often partially occluded by their own body parts, other people, furniture, or other objects; or parts of the body are outside the image. Self-occlusion is covered in all three databases. Human3.6M is comprised of more self-occlusions than the other datasets (and also some occlusions by chairs), because it includes many occlusion-causing actions such as sitting, lying down, or bending down. Occlusions by other people are common in Panoptic’s multi-person sequences. Additionally, parts of the bodies are quite frequently outside of the cameras’ field of view in Panoptic;
- Subject appearance: Human3.6M and especially HumanEva-I suffer from a low number of subjects, which restricts variability in body shapes, clothing, hair, age, ethnicity, skin color, etc. Although Panoptic includes many more and quite diverse subjects, it may still not sufficiently cover the huge diversity of real-world human appearances;
- Cameras: In-the-wild data are recorded from different viewpoints with varying resolutions, noise, motion blur, fields of view, depths of field, white-balance, camera-to-subject distance, etc. Within the three databases, only the viewpoint is varied systematically, and the other factors are mostly constant. With more than 500 cameras, Panoptic is the most diverse regarding viewpoint (also using three types of cameras). In contrast to the others, it also includes high-angle and low-angle views (down- and up-looking cameras). If only a few cameras are used, as in Human3.6M and HumanEva-I, there may be a bias in the body poses, because people tend to turn towards one of the cameras (also see [86] on this issue);
- Actions and poses: HumanEva-I and Human3.6M are comprised of the acted behavior of several action categories, whereas the instructions in Human3.6M allowed quite free interpretation and performance. Further, the actions and poses in Human3.6M are much more diverse than in HumanEva-I, including many everyday activities and non-upright poses such as sitting, lying down, or bending down (compared to only upright poses in HumanEva-I). However, some of the acted behavior in Human3.6M used imaginary objects and interaction partners, which may cause subtle behavioral biases compared to natural interaction. Panoptic captured natural behavior in real social interactions of multiple people and interactions with real objects such as musical instruments. Thus, it should more closely resemble real-world behavior;
- Annotated skeleton joints: The labels of the datasets, the ground truth joints provided, differ among the datasets in their number and meaning. Most obviously, the head, neck, and hip joints were defined differently by the dataset creators. In Section 4.1, we discuss this issue in detail and propose a way to handle it.

Although all the datasets have been and still are very useful to advance the state-of-the-art, we expect that many of these datasets’ biases will degrade real-world performance in 3D human pose estimation. As all the datasets were sampled from the real world, we used training and testing with different databases as a surrogate for roughly estimating the expected in-the-wild performance. Such cross-database evaluation is a common practice or a targeted goal in many other domains of computer vision [25,97,98,99,100,101,102].

Some of the biases, such as lighting, background, as well as subject ethnicity, clothing, and hair, only affect the images, but not the position of body joints. A limited diversity in these factors may be acceptable in 3D pose estimation datasets, because it is no problem for training a geometry-based approach that estimates the 3D pose from the 2D joint positions, given the used 2D pose estimation model has been trained with a sufficiently diverse 2D pose estimation dataset. Other factors, especially cameras and poses, heavily influence the position of body joints and must be covered in great diversity in both 2D and 3D pose datasets.

## 4. Methods

### 4.1. Joint Harmonization

As mentioned before, the skeleton joint positions provided in the datasets differed in their number and definition. To be able to conduct cross-dataset experiments, we selected a common set of 15 joints based on HumanEva-I. One keypoint was the central hip joint, which is the origin of the local body coordinate system, i.e., it is always at (0, 0, 0). Thus, we excluded it from the training and error evaluation. The remaining 14 joints are listed in Table 5. The first problem we faced was that there was no head keypoint in Panoptic, because this has not been annotated in the MS COCO dataset [96], which is used for training OpenPose [27,31] and other 2D pose estimators. However, there are MS COCO keypoints for the left and right ear. We calculated the center of gravity of these two points (Number 17 and 18 in Panoptic and OpenPose) in order to get a keypoint at the center of the head.

**Figure 2:**
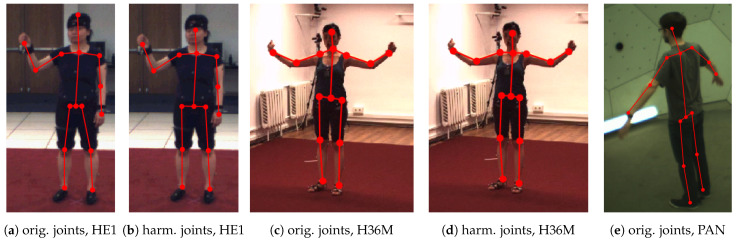
Examples showing the skeleton of HumanEva-I (HE1) and Human3.6M (H36M) before (“original” = orig.) and after the harmonization (harm.) of the head, neck, and hip joints. Panoptic (PAN) was used as the reference for harmonization.

After this step, we had keypoints for all the joints listed in Table 5. However, there were still obvious differences in some of the joints’ relative placements, as illustrated in Figure 2a,c,e. The different skeleton joint definitions introduced systematic errors into the cross-dataset experiments. To counter these effects, we harmonized the joint positions, using Panoptic (and thus the MS COCO-based joints) as the reference. This facilitated combining the 3D pose estimation with MS COCO-based 2D pose estimators, which is a promising research direction, and comparing future results with ours.

We adjusted the obvious differences in the head, neck, and hip positions in the HumanEva-I (HE1) and Human3.6M (H36M) datasets: (1) the head joint was moved to in between the ears in both HE1 and H36M; (2) the neck was placed between the shoulders in the H36M; and (3) the hip width (distance between the left and right hip keypoints) was expanded in HE1 and reduced in H36M.

To be more precise, the positions of the head and hip joints of the HE1 dataset were harmonized as follows: To move the head closer to the neck, we multiplied the direction vector between the neck and the head joint by a factor of 0.636. We calculated the factor from the ratio of the means of the neck-to-head length of the HE1 (316.1 mm) and the Panoptic (PAN) test datasets (201.1 mm). The hip joint distance was increased from the common center point by a factor of 2.13, again based on scaling the direction vector by the ratio of the mean distances (PAN 205.2 mm, HE1 96.3 mm). Figure 2a,b illustrates the effect of our adjustment.

The joint harmonization of the H36M dataset changed the position of the neck, head, and hip joints. The neck joint, which was defined at a higher position than in the other datasets, was moved to the center between the shoulder joints. To move the head point closer to the neck, we multiplied the direction vector between the repositioned neck joint and the head joint by a factor of 0.885. The factor was calculated from the ratio of the means of the neck-to-head length of the H36M (227.3 mm) and the PAN test datasets (201.1 mm). The hip joint distance was reduced from the common center point to 0.775 of the original value, based on the mean distances (PAN 205.2 mm, H36M 264.9 mm). Figure 2c,d illustrates the effect of the adjustment.

We provided the Python source code for harmonizing the joints at https://github.com/mihau2/Cross-Data-Pose-Estimation/ (accessed on 27 May 2021).

### 4.2. Scale Normalization

People differ in their heights and limb lengths. On the one hand, this is a problem for 2D-image-based 3D pose estimation, because, in the general case, the real height and limb lengths of a person (as well as the distance from the camera) cannot be measured from a single 2D image; therefore, accurate estimation of 3D joint positions is only possible up to an unknown scaling factor. Nevertheless, most state-of-the-art methods train their relative pose estimation models in a way that forces them to implicitly predict this scale, because they train the models to predict 3D joint coordinates, which implicitly contain the overall scale and the body proportions. This imposes a burden that encourages the models to learn dataset-specific heuristics, such as the height of individual subjects, the mean height of the subjects, the characteristics of the camera used, or the expected height/depth depending on the position and/or size of the person in the image. We expect that this way of training worsens generalization to in-the-wild data and in cross-dataset evaluations. On the other hand, knowing the scaling factor (the real height and limb lengths of the person) is not necessary for many applications that only require relative joint positions. Normalizing the joint positions from absolute to relative coordinates is common practice. We went a step further and proposed to normalize the scale of the skeletons, in order to remove the (often unnecessary) burden of predicting the scale and to improve the cross-dataset performance.

The absolute joint coordinates $p_{i}$ of each pose sample were normalized individually based on the skeleton’s relative joint positions in relation to the center hip point $p_{0}$, which was in the origin of the local coordinate system. We quantified the scale *s* by calculating the mean of the Euclidean distances between the origin and all *N* joint positions:

(1) $$s=\frac{1}{N}\sum_{i=1}^{N}∥p_{i}-p_{0}∥$$

Afterwards, we resized the skeleton by dividing all joint position coordinates by the scale, yielding a normalized scale of 1. The normalized joint positions ${\hat{p}}_{i}$ were calculated as follows:

(2) $${\hat{p}}_{i}=\frac{1}{s}\left(p_{i}-p_{0}\right)$$

This normalized all poses to a similar coordinate scale. This transformation was applied individually in both the 3D target data (with $p_{i}\in R^{3}$) and the 2D input data (with $p_{i}\in R^{2}$).

### 4.3. Baseline Model and Training

We performed our experiments with the “Baseline” neural network architecture proposed by Martinez et al. [26]. We decided to use an existing method rather than developing a completely new approach, because the focus of our work was on cross-dataset evaluation and proposing improvements that can be applied in many contexts. The approach by Martinez et al. did not rely on images, but mapped 2D skeleton joint positions to relative 3D joint positions, which is also called “lifting”. Thus, it can be combined with any existing or future 2D body pose estimation method. This way, the results can benefit from advances in 2D pose estimation, which are faster than in 3D pose estimation, because in-the-wild 2D pose datasets are much easier to create than their counterparts with 3D ground truths. Other advantages include: (1) The approach is independent of image-related issues, such as lighting, background, and several aspects of subject appearance, which are covered with great diversity in 2D pose datasets. By decoupling the 2D pose estimation from the “lifting”, we avoided overfitting the 3D pose estimation to the quite restricted diversity of the 3D pose datasets regarding lighting, background, and subject appearance. (2) The approach allowed augmenting the training data by creating synthetic poses and virtual cameras, which can massively increase the variability of the available data and lead to better generalization. (3) No images were needed, so additional sources of training data may be exploited, such as motion capture data recorded in sports, biomechanics, or entertainment. (4) The source code is available. Therefore, it is easy to reproduce the results, apply the method with other data, and start advancing the approach.

The architecture by Martinez et al. [26] was a deep neural network consisting of fully connected layers and using batch normalization, ReLU, dropout, and residual connections. The first layer maps the 2D coordinates (2n=28 dimensions) to a 1024-dimensional space. It is followed by two residual blocks, each including two fully connected layers. Finally, there is another linear layer that maps the 1024-dimensional space to the 3n=42-dimensional 3D coordinate output.

The model, training, and testing were implemented in the TensorFlow2 deep learning framework using the Keras API. The networks were trained with the Adam optimizer, minimizing the mean squared error loss function. The training set was separated into training and validation data with a 90/10% split, and the training data were shuffled before each epoch. We used a batch size of 512 and a dropout rate of 0.5. The training of each neural network started with a learning rate of $10^{-3}$, which was reduced during the training by a factor of 0.5 if the loss on the validation set did not decrease for 3 epochs. The training was stopped if the validation loss did not decrease for 10 epochs or the learning rate was reduced below $10^{-6}$. The model with the lowest validation loss was saved for testing.

### 4.4. Anatomical Pose Validation

We proposed an optional pose validation step, which assessed the predicted poses using the constraints of human anatomy. The human body is usually symmetrical regarding the length of the left and right extremities and has, according to Pietak et al. [103], stable ratios regarding the lengths of the upper and lower limbs with little variation between individuals.

For every pose, the ratios of each upper and lower extremity, as well as its left and right counterpart were calculated. The ratios were measured as the difference in length in %, based on the shorter of the two compared limbs. Therefore, a ratio of 2:1 and 1:2 would both result in a difference of +100%. If one of the 8 calculated ratios was greater than 100%, the pose was rejected by the validation.

The effect of this approach is analyzed in Section 5.7. All the other experiments were conducted without applying this validation step, because it led to the exclusion of rejected poses from the error calculation and thus limited the comparability of the error measures (which may be based on different subsets of the data).

### 4.5. Use of Datasets

**Figure 3:**
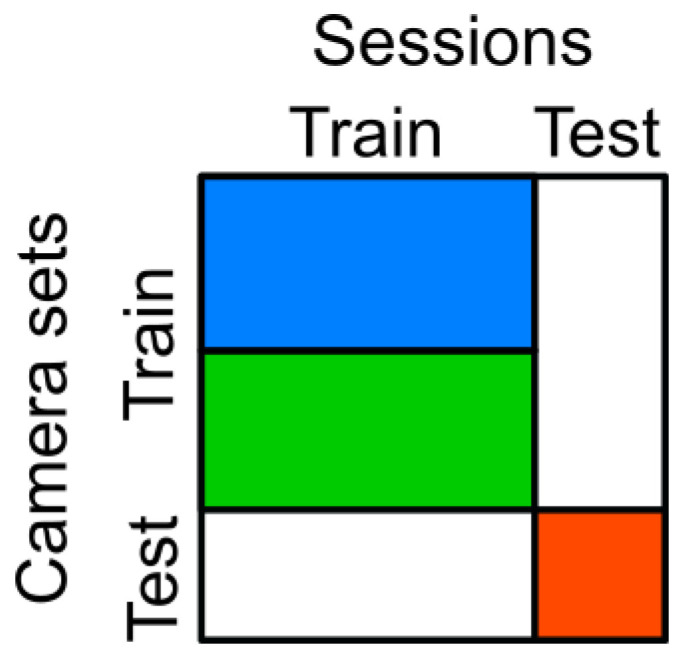
Separation of the training and test sets for the subject sessions and camera sets (blue: reduced training camera set; blue and green: full training camera set; red: test camera set; white: unused data).

**Figure 4:**
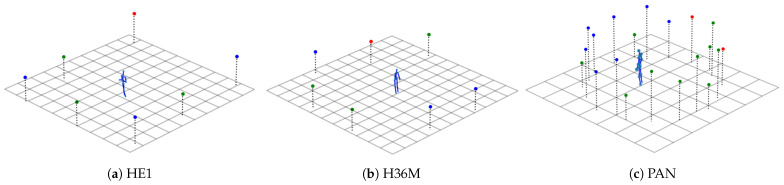
Camera positions and an example pose for the used datasets (grid at z = 0 with 1 meter cell size; blue: reduced training camera set; blue and green: full training camera set; red: test camera set).

Each dataset was split into training and test data based on the sessions/subjects and cameras, as illustrated in Figure 3. No single camera or session was used for both the test and training set. Although parts of the datasets were unused, we selected this way of splitting because our focus was to measure the generalization across subjects, camera viewpoints, and datasets rather than reaching the highest in-dataset performance. The cameras were assigned to the test set and the reduced and full training set, as illustrated in Figure 4 and detailed below. Further, because the number of cameras was quite low in Human3.6M (only 4), we generated synthetic camera views as described in Section 4.5.2. After the main split, the training data was further randomly split into 90%, which were used as the actual training set by the optimizer, and 10%, which were used as the validation set.

#### 4.5.1. Dataset Split Details

For HumanEva-I (HE1), Subjects 0 and 1 were used as the training set and Subject 2 as the test set. The camera “BW1” was used for the test set. We used the other black-and-white and color cameras for the training set. The reduced camera set only contained the black-and-white cameras. For the evaluation using the OpenPose 2D joint positions, we used all videos of Subject 2 that contained the corresponding video and motion capture data. This reduced the OpenPose test dataset, in comparison to the standard test set, because only a subset of the sessions included both motion capture and video files.

For Human3.6M (H36M), Subjects 1, 5, 6, 7, and 8 were used for the training set and Subjects 9 and 11 for testing. We used Camera 3 as the test camera. Cameras 0, 1, and 2 were the reduced camera training set. The full camera training set contained Cameras 0, 1, and 2 and their modified synthetic copies (see Section 4.5.2). For the evaluation using the generated OpenPose 2D joint estimations, we used all videos of Subjects 9 and 11.

For Panoptic (PAN), the *Range of Motions* sessions (sequence names: 171026_pose1, 171026_pose2, 171026_pose3, 171204_pose1, 171204_pose2, 171204_pose3, 171204_pose4, 171204_pose5, 171204_pose6) were used for testing and all other sessions for training. Of each panel, we only used VGA Camera No. 1. The cameras on Panels 9 and 10 were used for testing. The cameras on Panels 1–8 were the reduced camera training set, and the cameras on Panels 1–8 and 11–20 were the full training set.

Table 6 shows the resulting sample sizes for the different databases and successfully mapped OpenPose (OP) samples.

#### 4.5.2. Virtual Camera Augmentation

The H36M dataset was recorded with only 4 cameras. In order to make the ratio of training and test cameras in the databases more similar, three more camera were added. For this purpose, we virtually copied the training Cameras 0, 1, and 2 by rotating their extrinsic camera parameters by 90° around the world coordinate center in the middle of the recording space without changing the intrinsic camera parameters. This can be seen in Figure 4b, where the blue points represent the original training camera positions, and the newly created cameras are shown in green.

### 4.6. Implementation Details

First, the original 3D pose data in the world coordinate space were loaded. If a pose contained a non-valid joint position, usually (0, 0, 0), the pose was discarded. Further, we used the jointwise confidence score provided in the PAN dataset to remove unreliable data. If the score of any of the 14 used joints was <0.1, we discarded the corresponding pose. Next, the 3D pose data were transformed to the camera coordinates for each camera of the used set.

If *scale normalization* was applied, the scale of the 3D joint positions was normalized to a mean distance of 1 from the center of the hips. After that, the pose was repositioned to the camera coordinates (0, 0, 50) for the projection step. All pose transformations described in this section used the center of the hips as the reference point.

In the next step, the 3D pose was projected onto the camera 2D image plane including the distortion parameters. Poses with at least one joint outside the projected camera image (1000×1000 px in H36M and 640×480 px in HE1 and PAN) were discarded. This was necessary due to the nonlinear components in the distortion model, which could result in extremely outlying projected points in the image plane if the 3D joint positions were not in the original image frame for which the distortion parameters were calibrated. Next, an additional pose validation step was performed. The limb lengths (for left and right: upper arm, lower arm, upper leg, lower leg, shoulder-to-neck; also the hip width and neck-to-head distance) were calculated once using the original joint descriptions for every database on its complete training set. From that data, the mean length μ and standard deviation σ of the noted limbs were determined. Irregular poses, where at least one limb length deviated more than 3σ from μ, were discarded in the full and reduced training set. These two data validation checks were only done for the projection with the original joint descriptions and without the use of *scale normalization*, to keep the differently processed datasets comparable. The validity status for each sample was saved and applied if the *scale normalization* and/or the harmonized joint descriptions were used, to ensure the same subset of samples was used regardless of the preprocessing steps.

If the *scale normalization* was applied, then the 2D joints of the pose were also normalized to a mean distance of 1 to the center of the hips, and the pose was moved to (0, 0). The 2D poses in the image coordinates were used as training inputs, and the 3D poses in world coordinates, moved to (0, 0, 0), were used as training targets. The data were normalized to a mean of 0 and a standard deviation of 1 for every net input and output channel.

For the evaluation of a model, the 2D inputs of the test dataset were normalized with the models’ normalization values calculated on the training set, and the resulting prediction outputs were denormalized analogously. If *scale normalization* was used, the output pose was first scaled up to the scale of the ground truth pose before calculating the joint errors.

## 5. Results

In this section, we summarize the results of our cross-dataset and in-dataset evaluation. All experiments were repeated five times, that is each reported result was the average performance of five independently trained models. The error was calculated as the mean of the sum of all joint Euclidean distances between the output and the corresponding ground truth pose in mm.

We calculated two error types for the evaluation: The first was a no-alignment error, where the data of the predicted output pose were not post-processed and directly compared to the relative 3D ground truth pose, with the center of the hips at (0, 0, 0). The *no-alignment* error was used for most of the results. Second, we calculated the *Procrustes* error, where the output pose was moved, scaled, and rotated, minimizing the joint distances between the prediction and ground truth. Some *Procrustes* error values are presented in Table 12 for comparison with the no-alignment errors. The other *Procrustes* error tables for the presented data can be found in the Supplemental Materials.

If not explicitly mentioned otherwise, the results reported in the following were obtained with harmonized joints and the full camera set.

The prediction speed on the trained models was tested using an NVIDIA GeForce RTX 2080 TI graphics card. A batch with a size of 256 samples was calculated in around 30 milliseconds, which would result in 8533 pose estimations per second. The proposed model can therefore calculate 3D poses from 2D points in real time.

### 5.1. Joint Harmonization

Table 7 shows the mean and standard deviation of the errors for the evaluation over all datasets with and without joint harmonization. All entries in a row share the same training database; those in a column share the same test database. On the main diagonal are the in-database errors, which were significantly lower than the cross-database error (off the main diagonal). This difference showed the presence of dataset biases and their negative effect on cross-dataset generalization

The joint harmonization improved the results significantly from an overall mean error of 133.7 mm to 120.0 mm (p=0.040, paired *t*-test). The impact differed among the individual training and test dataset combinations. As to be expected, the estimation error was mainly reduced in the cross-database results, where it was decreased by up to −29%. The greatest effect can be seen for HE1, which was the smallest dataset and whose joint definition deviated most from those of the other datasets.

The high absolute errors of the models trained with the HE1 were especially prominent in the ankle and knee joints. The errors can be attributed to the low diversity of poses in HE1, which did not include wide arm movements and no non-standing poses, which however were very common in H36M and PAN.

### 5.2. Number of Cameras

We compared the estimation error for the full camera set with a reduced camera set. For this purpose, the amount of used cameras was halved. Details about the used camera sets and their placement can be found in Section 4.5 and Figure 4.

Table 8 shows the results. The use of more cameras, and therefore more viewpoints and pose samples, changed the individual testing errors by in between 6.8% and −28.8%. Overall, the mean error decreased from 132.6 mm to 120.0 mm, which was a statistically significant difference (p=0.031 in a one-sided paired *t*-test). The increase in the number of cameras had a positive impact on the testing results when training with the HE1 or H36M dataset, which both only had three camera views in the reduced camera set, with changes in the error of −5.1% up to −28.8%.

### 5.3. Scale Normalization

Table 9 shows the mean error and the standard deviation for the evaluation with and without scale normalization. Scale normalization significantly decreased the pose estimation error, from on average 120.0 mm to 90.1 mm (p=0.015 in a one-sided sample-paired *t*-test). For the in-database evaluation, the error decreased between −13.2% and −24.6%. Cross-database testing resulted in even bigger reductions up to −42.9%.

The results of the models trained on HE1 and H36M and tested on the PAN dataset showed less improvement or even a worse result when using scale normalization. This error increase can be attributed to the test samples with a low camera viewing angle, which was not contained in the HE1 and H36M datasets.

Interestingly, the scale normalization error when training on PAN and testing on HE1 decreased below the in-database error of HE1. The training set of PAN was larger and more diverse than that of HE1, which helped the cross-dataset generalization outperform the in-dataset generalization in this case.

**Figure 5:**
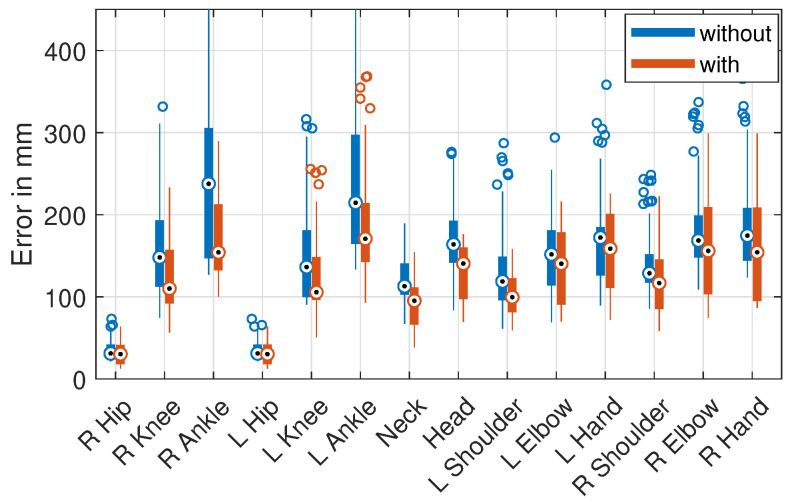
Jointwise error of all cross-dataset results with and without scale normalization. Box plot with median (circle with dot), 1’st/3’rd quartile (bottom/top of thick bar), and outliers (circles, default settings of MATLAB 2017b).

Figure 5 shows the jointwise errors with and without scale normalization of only the cross-database evaluation as a box plot. The median error decreased for all joints, most for the leg joints. Most of the high-error outliers occurring with the original representation disappeared when using scale normalization.

In order to illustrate the need for scale normalization, we calculated the scale differences without the normalization as the ratio of the Frobenius norms, of all joints, of the predicted and ground truth poses, after moving the centroid of the poses to (0, 0, 0). Table 10 shows the scale differences for the test sets. Several systematic prediction errors of up to 16% can be seen in the scale ratios, especially in the cross-dataset experiments when testing on HE1 and H36M. We found an interesting in-database result (main diagonal) with HE1: The scale of the predicted HE1 test poses (0.89) was significantly smaller than the absolute scale of the ground truth, while the PAN (1.0) and H36M (0.99) model predicted their own scale from their training data with greater accuracy.

This difference in the size of the ground truth poses can be attributed to the distances between the cameras and the recorded subjects. The camera positions in the HE1 were set up in a rectangle of around 8 × 9 m with a capture space of 2 × 3 m in the center of that. Our randomly chosen test camera was at one of the corners and therefore one of the most distant cameras in the dataset. The H36M dataset had its cameras in a 5 × 10 m setup and used a capture space of 3 × 4 m. We virtually copied and rotated the three training cameras so that the cameras were positioned close to circularly around the subjects. The PAN dataset had a capture space with diameter of 5 m in which the subjects could act freely, but due to the curvature of the dome and the constraint that the pose had to be fully captured in the camera view, only a limited range of distances could be used for training.

**Figure 6:**
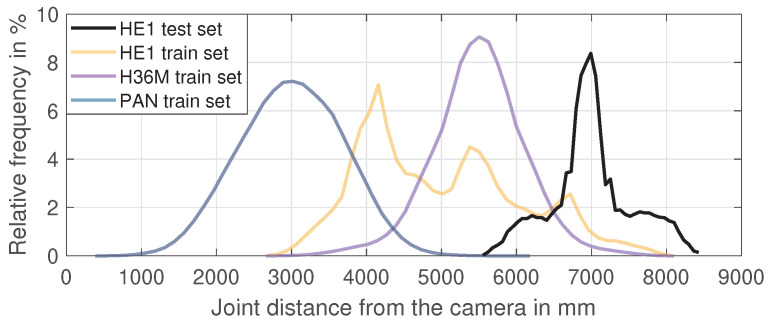
Relative distribution of all joint-to-camera distances for the HE1 test set and all training sets (full camera set).

Therefore, the positioning of the cameras and the capture spaces led to different distances from the recorded subjects and systematic differences in the pose scale in the training data. Figure 6 shows the relative distribution of all joint-to-camera distances for some of the training and test datasets. It can be seen that the training poses of all datasets and testing poses of HE1 differed strongly in the distance to the cameras, which probably resulted in the failure to predict the true scale of the presented 2D pose. Other factors that can lead to this effect are the camera field-of-view/focal length, the camera resolution, and systematic biases in the body size of the subjects. The presence and effect of such dataset biases illustrate the importance of scale normalization for improving the cross-dataset and in-the-wild performance of 3D pose estimation.

### 5.4. Multi-Database Training

In order to improve the cross-dataset generalization, we tried to increase the diversity in the training data by combining datasets. We used a leave-one-out approach for the training and testing, that is we always left out one database for cross-database testing and used the other two for training. The training sets were combined by concatenating the data (and new normalization parameters for the nets inputs and outputs were derived).

Table 11 shows the generalization errors with and without scale normalization. Scale normalization improved the pose estimation error in the multi-database training, with a value of p=0.003 in a paired t-test. For cross-database training and test cases, the error decreased between −0.6% and −50.1%. Similar to the single-database training in Table 9, the effect was bigger on the HE1 and H36M test set than on PAN. The error for the cases, in which the model was tested on one of the training databases, decreased between −10.8% and −41.5%.

In Table 11, single-database training results are added for easier comparison to multi-database. When testing on the HE1 dataset, combining H36M and PAN for training improved the cross-database results from 67.3 mm (PAN only) to 64.9 mm, which was significantly below using HE1 for training (69.2 mm). Further, combining HE1 and PAN for training reduced the error slightly below using PAN only. Apart from that, the multi-database training did not reduce the test errors in comparison to single-database training; Training with the bigger dataset alone achieved a similar or slightly better result than training with the combination of two datasets.

### 5.5. OpenPose Evaluation

In order to test the generalization of the 3D pose estimation with a widely used 2D pose estimator, we conducted experiments with OpenPose [31].

First, the test set videos of the HE1 and the H36M datasets were processed with OpenPose. The videos of the Panoptic database could not be obtained on several occasions, due to availability issues with the host file server. The obtained 2D joint coordinates were used as inputs for the trained models to predict 3D joint positions, which were compared to the ground truth pose data. Note that the models were not fine-tuned with points provided by OpenPose. A noticeable difference between the two OpenPose datasets was the underlying image quality. While the HE1 was recorded at 640×480 px, the H36M dataset had a higher resolution of 1000×1000 px and better image quality. The video frames and motion-capture joint poses were synchronized for the OpenPose evaluation. The synchronization was manually corrected for the HE1 with an offset of 10 frames. The 3D pose evaluation error for every frame was calculated to the timewise closest motion-capture pose if that corresponding pose was valid.

Table 12 shows the test results for the OpenPose (OP) data and, for better comparison, the standard evaluation results. The errors are given for the *no-alignment* case and after the *Procrustes* alignment. As in the previous sections, the test results were generally better when training and testing with the same dataset, except for HE1. When testing on HE1 and HE1 (OP), the cross-database training on PAN outperformed the in-database training on HE1 in both the no-alignment and Procrustes error. On HE1 (OP) with Procrustes error, also, cross-dataset training with H36M performed better than in-dataset training with HE1.

The *no alignment* error for the H36M (OP) test dataset was, excluding the HE1-trained models, consistently around 50 mm higher compared to the projected H36M data. This increase was evenly distributed over most of the joints, with the exception of the hip joints, for the models trained on both the H36M itself and the PAN datasets. The models trained on HE1 achieved an error reduction on certain joints (R knee, R ankle) and increased in the others, which was probably due to the lack of training data and the higher error rates to begin with. Similar effects can be seen for the results of the HE1 (OP) dataset, where the error increase was also distributed over all joints for all test cases, with slightly lower error increases for the hip, neck, and shoulder joints.

The *Procrustes* calculation minimized the errors in the scaling, rotation, and positioning of the skeleton. Therefore, the errors were smaller than without this alignment step in all cases. Analogous to the *no alignment* error, the results for the testing on the H36M (OP) dataset were, excluding the HE1 trained models, consistently around 20 mm higher compared to the projected H36M data. The results for the HE1 (OP) dataset were around 40 mm higher than for the projected HE1 data. For the projected test datasets, the error reductions for the same training and test database cases were between −16.4% and −25.6%, and the the cross-database results improved by up to −45.5% The absolute errors for the pose estimation were reduced to a range between 28 mm and 61 mm using the bigger training datasets (H36M, PAN) and 105 mm for the smaller HE1.

**Figure 7:**
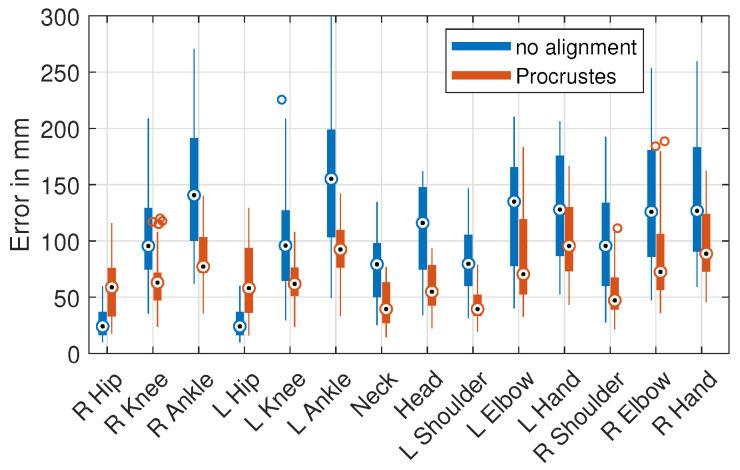
Jointwise no-alignment and Procrustes error of all single-database training results. Box plot with median (circle with dot), 1’st/3’rd quartile (bottom/top of thick bar), and outliers (circles, default settings of MATLAB 2017b).

The *Procrustes* error changes of the individual joints are shown in Figure 7. It can be seen that rotation and repositioning during the *Procrustes* optimization increased the error in the hip joints, but decreased the error for all other joints. The effect increased with the distance to the skeletal root between the hips, because the joints further away from the center tended to have a greater impact on the Procrustes distance and minimization.

### 5.6. Rotation Errors

Due to the separation of the cameras into training and test sets, the test camera viewpoints were not used for the training and were novel to the models. This often led to skeleton predictions with rotation errors. We calculated the rotation error from the Procrustes alignment as the magnitude of the minimal rotation in 3D space needed to minimize the joint distances between the ground truth and prediction. Table 13 shows the rotation errors for the OpenPose and projected test sets, using both camera sets and with or without scale normalization.

The rotation error generally decreased with the addition of new camera positions, which we saw when comparing the first part of the table (reduced cam set) with the second part (full cam set). The effect was especially strong (−6.6${}^{\circ }$) when training on HE1 and testing on PAN.

The additional use of scale normalization decreased the error for most of the combinations even further, up to −9.7° and −9.3° for the HE1 and H36M cross-dataset evaluations. The PAN-trained models also had better rotation accuracy with the cross-database test results decreasing from −4.4° to −9.8°. The effects were smaller (−1.2° HE1 and −0.7° H36M) or even slightly worse (+0.6° PAN) for in-database training and testing. An outlying increase of the rotation error can be seen for the HE1 trained models, when evaluated on the PAN dataset. This was probably due to the introduction of bigger camera-to-pose view angles by the repositioning of the poses before the 3D to 2D projection.

### 5.7. Anatomical Pose Validation

Table 14 compares the pose estimation results with and without the anatomical pose validation that we proposed in Section 4.4. The validation approach successfully identified many wrongly estimated poses, which was revealed by the decreasing error in all tested database combinations.

For the testing on the projected ground truth data (HE1, H36M, and PAN), the decreases were smaller for in-database, with decreases from −0.5% to −2.5%. Bigger improvements can be seen in the results for the cross-database testing. The error rates decreased here from −1.3% to −9.3%.

The biggest impact of the pose validation was on the models trained with the HE1 dataset. It had the biggest error reduction, and up to 20.7% of the poses were rejected, while the rate for the other datasets was between 0.3% and 3.3%. Many poses that occurred in H36M and PAN test data were not part of the small HE1 dataset, e.g., HE1 only contained upright poses and only a limited range of arm and leg movements. Training with this dataset resulted in poor generalization to completely unseen poses, leading to many anatomically impossible skeletons. The other two datasets reached a lower cross-database pose rejection in the range from 1.1% to 3.3%, which showed better generalization.

All datasets had high pose rejection rates on the HE1 (OP) testing set. This effect was not present for the H36M (OP) dataset, where only the HE1-trained models showed a higher pose rejection rate, which was similar to the rate for the ground truth projection H36M dataset. This correlated with the low sample size of the training data and poor video quality of the HE1 dataset, which led to higher pose errors for all models.

## 6. Discussion

In this article, we conducted cross-dataset experiments and discussed dataset biases as a step towards better cross-database generalization and in-the-wild performance of 3D human pose estimation systems.

The used datasets, HumanEva-I, Human3.6M, and Panoptic datasets, differed in their ground truth skeleton joint definitions, which impeded using these datasets together. Thus, we proposed a joint harmonization approach that facilitated cross-dataset experiments and reduced the biases among the datasets. In-the-wild performance would benefit from unifying the ground truth of additional datasets. However, a limitation of our approach was that it needed to be parameterized manually for each new dataset. For future works, it may be promising to develop generalized, automatic, and more accurate harmonization methods for post-processing existing datasets and to agree on a standardized skeleton joint model for collecting new datasets.

We analyzed the impact of the number of camera viewpoints used for the training. For databases with a small number of cameras such as H36M and HE1, adding more cameras improved the pose estimation significantly for in-database and cross-database evaluation. This showed that a certain coverage of viewpoints was needed for good generalization. With approaches that lift 2D poses to 3D poses, such as the one of Martinez et al. [26], datasets may be augmented by projecting the 3D ground truth to new virtual cameras, as was tested on the H36M dataset, improving the evaluation error up to −14%.

Many prior works expected the pose estimation model to learn the correct 3D scale from single-image 2D data, which is an impossible task in the general case. This imposed a burden that encouraged the models to learn dataset-specific heuristics and, as a consequence, to overfit to the dataset. We showed that the used databases were biased regarding the parameters, positions, and distances of the used cameras, which resulted in systematic scale errors in the output of the trained poses. Our proposed *scale normalization* step reduced the pose estimation error on the test datasets significantly, in 17 of 18 test cases and in the best case by more than −50% (see Table 9 and Table 11). We investigated the one case in which the scale normalization decreased the performance. In this case, the repositioning of test poses in the preprocessing step increased the relative rotation of the pose to the camera, which led to higher prediction errors because these rotations were not present in the training dataset. However, this weakness could be compensated by augmenting the training dataset using virtual cameras with additional viewing angles, as mentioned above. Another limitation of the presented scale normalization approach is that the effects of camera distortions cannot be trained, because the position and scale are normalized in both 2D and 3D space. This error was not relevant in comparison to other factors in our experiments, but could become an issue for cameras with a very wide field of view.

Several of the dataset biases could be compensated in future works by adding virtual cameras, as described in Section 4.5.2, with various camera elevations, angles, and distances. We see this as a promising and more general augmentation approach for all available pose datasets. This approach could generate more training data for camera-to-subject distances and view angles with variation of the extrinsic camera calibration parameters. More camera types can also be added by variation of the intrinsic camera parameters, such as the focal length, to create data with different angles-of-view and enable better generalization. Additionally, this idea may be used with arbitrary motion capture data, including data for which no images are available, but probably requires advancing the proposed harmonization approach as mentioned above.

The presented anatomical pose validation achieved a high rate of pose rejection for the small HE1 dataset, catching malformed poses originally not contained in the training dataset. It also identified many invalid poses predicted with OpenPose from low-quality video. Most of the rejected poses had big shifts in the depth component (distance from the camera) of one or multiple joints, probably because there was no similar pose in the training set. Next to such a validation step, a promising alternative direction for all future works would be to include anatomical constraints in the model training to avoid such errors in the first place, e.g., as proposed in [40,51].

The evaluated multi-dataset training could not consistently improve the results compared to single database training, probably due to the big differences in the sample size of the used datasets (by a factor of approximately 10 to 40). A combination of databases is probably most beneficial if the datasets contain different poses and motions that can add new information, while more camera viewing angles may be created artificially, as stated above.

The prior work that is most similar to our work was published recently by Wang et al. [86]. They systematically examined the differences among existing pose datasets and their effect on cross-database evaluation. However, compared to our work, they focused on the systematic differences of camera viewpoints and conducted their experiment with another set of databases. Quantitative comparison to other works is difficult, because our evaluation protocol was designed for cross-dataset experiments that have not been published before. Nevertheless, the improvement by methodological advancements can be measured in comparison with the approach of Martinez et al. [26], which we used as the starting point. Table 15 shows that the proposed modifications (joint harmonization, scale normalization, and virtual camera augmentation (tested when training with H36M)) improved generalization across subjects, camera viewpoints, and datasets. The proposed anatomical pose validation (APV) reduced the error further. Joint harmonization, scale normalization, and APV can be applied with other 3D pose estimation approaches, and we see this as a promising direction for improving generalization. Virtual camera augmentation can be applied for all 2D to 3D pose lifting approaches, which may easily benefit from motion capture data and synthesized data and avoid overfitting to image-related dataset biases.

As a promising direction for improving the cross-database performance (and testing of the proposed approaches), we suggest a multi-task training combining in-the-wild 2D datasets with 3D datasets, integrating a pretrained 2D-to-3D pose lifting network. Further, a logical advancement of our work is evaluating cross-database performance with additional datasets, especially new “in-the-wild” datasets, in order to gain additional insights about dataset biases and about how to improve 3D pose estimation so that it works well on arbitrary data.

## Figures and Tables

**Table 1:** Mean per-joint position error (MPJPE) for state-of-the-art approaches on H36M.

Method (Reference)	MPJPE (mm)	Method (Reference)	MPJPE (mm)
Ionescu et al. [20]	162.1	Habibie et al. [38]	65.7
Pavlakos et al. [39]	115.1	Zhou et al. [40]	64.9
Chen and Ramanan [41]	114.2	Sun et al. [42]	64.1
Zhou et al. [43]	113.0	Luo et al. [44]	61.3
Tome et al. [45]	88.4	Rogez et al. [46]	61.2
Martinez et al. [26]	87.3	Nibali et al. [47]	55.4
Pavlakos et al. [48]	75.9	Luvizon et al. [49]	53.2
Wang et al. [50]	71.9	Dabral et al. [51]	52.1
Tekin et al. [52]	69.7	Li et al. [53]	50.9
Chen et al. [54]	68.0	Lin and Lee [55]	46.6
Katircioglu et al. [56]	67.3	Chen et al. [57]	44.1
Benzine et al. [58]	66.4	Wu and Xiao [59]	43.2
Sárándi et al. [60]	65.7	Cheng et al. [61]	42.9

**Table 2:** Mean per-joint position error (MPJPE) for state-of-the-art approaches on the PAN dataset.

Method (Reference)	MPJPE (mm)
Popa et al. [62]	203.4
Zanfir et al. [63]	153.4
Zanfir et al. [64]	72.1
Benzine et al. [58]	68.5

**Table 3:** Mean per-joint position error (MPJPE) for state-of-the-art approaches on the HumanEva-I dataset.

Method (Reference)	MPJPE (mm)
Radwan et al. [65]	89.5
Wang et al. [50]	71.3
Yasin et al. [66]	38.9
Moreno-Noguer [67]	26.9
Pavlakos et al. [68]	25.5
Martinez et al. [26]	24.6
Pavlakos et al. [39]	18.3

**Table 4:** Quantitative comparison of the datasets.

	HumanEva-I	Human3.6M	Panoptic
Subjects	4	11	>100
Actions	6	15	many
Multi-person	-	-	✓
Recording duration	10 min	298 min	689 min
Cameras	7	4	>500
Total frames	0.26 M	3.6 M	>500 M
Skeleton joints	15	32	19

**Table 5:** Our joint definitions for the different datasets and OpenPose. The numbers in the table correspond to the joint number in the datasets’ original joint definition. The joints marked with * were repositioned in the harmonization process.

Joint	HumanEva-I	Human3.6M	Panoptic	OpenPose
R Hip	1 *	1 *	12	9
R Knee	2	2	13	10
R Ankle	3	3	14	11
L Hip	4 *	6 *	6	12
L Knee	5	7	7	13
L Ankle	6	8	8	14
Neck	7	13 *	0	1
Head	8 *	15 *	(17 + 18)/2	(17 + 18)/2
L Shoulder	9	17	3	5
L Elbow	10	18	4	6
L Hand	11	19	5	7
R Shoulder	12	25	9	2
R Elbow	13	26	10	3
R Hand	14	27	11	4

**Table 6:** Sample sizes in thousands of poses.

	Training Set		Testing Set
	Reduced	Full	
HE1	113	225	17.8
H36M	1169	2312	137.7
PAN	4131	9809	292.0
HE1 (OP)	-	-	1.7
H36M (OP)	-	-	52.1

**Table 7:** Errors with original vs. harmonized joints (no-alignment errors in mm, mean ± std. deviation).

Training Data	Test Data		
	HE1	H36M	PAN
	original joints (mean 133.7)		
HE1	95.9 ± 2.9	299.7 ± 9.5	148.8 ± 4.9
H36M	142.1 ± 3.9	67.6 ± 0.6	95.1 ± 3.2
PAN	166.7 ± 2.4	143.6 ± 1.2	43.9 ± 0.3
	harmonized joints (mean 120.0)		
HE1	91.7 ± 1.9	254.1 ± 5.8	125.4 ± 4.3
H36M	141.7 ± 3.8	67.0 ± 0.6	98.3 ± 2.2
PAN	117.8 ± 2.4	140.4 ± 1.3	43.7 ± 0.2
	mean error change		
HE1	−4.3%	−15.2%	−15.7%
H36M	−0.2%	−0.9%	3.4%
PAN	−29.3%	−2.3%	−0.6%

One-sided paired-sample *t*-test *p* = 0.040.

**Table 8:** Errors with the reduced vs. the full camera set (no-alignment errors in mm, mean ± std. deviation).

Training Data	Test Data		
	HE1	H36M	PAN
	reduced camera set (mean 132.6)		
HE1	96.6 ± 1.9	270.6 ± 11.5	176.2 ± 5.4
H36M	166.4 ± 7.7	75.1 ± 0.4	105.2 ± 5.5
PAN	129.5 ± 2.1	131.4 ± 0.7	42.2 ± 0.3
	full camera set (mean 120.0)		
HE1	91.7 ± 1.9	254.1 ± 5.8	125.4 ± 4.3
H36M	141.7 ± 3.8	67.0 ± 0.6	98.3 ± 2.2
PAN	117.8 ± 2.4	140.4 ± 1.3	43.7 ± 0.2
	mean error change		
HE1	−5.1%	−6.1%	−28.8%
H36M	−14.8%	−10.7%	−6.6%
PAN	−9.0%	6.8%	3.4%

One-sided paired-sample *t*-test *p* = 0.031.

**Table 9:** Error with and without scale normalization (no-alignment errors in mm, mean ± std. deviation).

Training Data	Test Data		
	HE1	H36M	PAN
	no scale normalization (mean 120.0)		
HE1	91.7 ± 1.9	254.1 ± 5.8	125.4 ± 4.3
H36M	141.7 ± 3.8	67.0 ± 0.6	98.3 ± 2.2
PAN	117.8 ± 2.4	140.4 ± 1.3	43.7 ± 0.2
	with scale normalization (mean 90.1)		
HE1	69.2 ± 0.7	170.3 ± 4.0	152.7 ± 2.7
H36M	86.0 ± 1.2	55.2 ± 0.5	89.2 ± 0.7
PAN	67.3 ± 1.0	83.1 ± 0.6	37.9 ± 0.4
	mean error change		
HE1	−24.6%	−33.0%	21.8%
H36M	−39.3%	−17.7%	−9.3%
PAN	−42.9%	−40.8%	−13.2%

One-sided paired-sample *t*-test *p* = 0.015.

**Table 10:** Mean scale ratios between ground truth and prediction without scale normalization.

Training Data	Test Data		
	HE1	H36M	PAN
	scale error (full cam set)		
HE1	0.89	1.09	0.97
H36M	0.90	0.99	1.01
PAN	0.84	0.90	1.00

**Table 11:** Error of multi-database training with and without scale normalization (no-alignment errors in mm, mean ± std. deviation).

Training Data	Test Data		
	HE1	H36M	PAN
	no scale normalization (mean 103.0)		
H36M + PAN	130.2 ± 2.9	103.8 ± 1.6	43.0 ± 0.3
HE1 + PAN	115.0 ± 1.3	143.0 ± 2.2	45.5 ± 1.6
HE1 + H36M	135.5 ± 1.2	75.1 ± 1.1	103.7 ± 4.3
	with scale normalization (mean 69.0)		
H36M + PAN	64.9 ± 0.5	63.0 ± 0.4	38.3 ± 0.3
HE1 + PAN	67.2 ± 0.7	83.2 ± 0.8	38.3 ± 0.7
HE1 + H36M	100.4 ± 1.2	62.6 ± 0.8	103.0 ± 2.1
HE1	69.2 ± 0.7	170.3 ± 4.0	152.7 ± 2.7
H36M	86.0 ± 1.2	55.2 ± 0.5	89.2 ± 0.7
PAN	67.3 ± 1.0	83.1 ± 0.6	37.9 ± 0.4
	mean error change		
H36M + PAN	−50.1%	−39.3%	−10.8%
HE1 + PAN	−41.5%	−41.8%	−15.9%
HE1 + H36M	−25.8%	−16.6%	−0.6%

One-sided paired-sample *t*-test *p* = 0.003.

**Table 12:** Error (no alignment vs. Procrustes) with OpenPose 2D joints (OP) and ground truth joint projection, with scale normalization (errors in mm, mean ± std. dev.).

Training Data	Evaluation Data				
	HE1 (OP)	H36M (OP)	HE1	H36M	PAN
	no alignment				
HE1	138.3 ± 1.3	184.4 ± 4.0	69.2 ± 0.7	170.3 ± 4.0	152.7 ± 2.7
H36M	151.3 ± 1.7	108.6 ± 0.7	86.0 ± 1.2	55.2 ± 0.5	89.2 ± 0.7
PAN	126.1 ± 1.2	130.8 ± 1.1	67.3 ± 1.0	83.1 ± 0.6	37.9 ± 0.4
	Procrustes alignment				
HE1	105.8 ± 0.6	109.5 ± 1.3	57.8 ± 0.7	105.0 ± 2.3	104.6 ± 1.9
H36M	103.1 ± 0.8	65.6 ± 0.6	61.4 ± 0.6	41.5 ± 0.2	48.6 ± 0.9
PAN	93.4 ± 0.7	71.9 ± 0.5	55.0 ± 0.8	55.4 ± 0.3	28.2 ± 0.3
	mean error change				
HE1	−23.5%	−40.6%	−16.4%	−38.3%	−31.5%
H36M	−31.8%	−39.6%	−28.6%	−24.8%	−45.5%
PAN	−26.0%	−45.0%	−18.3%	−33.3%	−25.6%

**Table 13:** Mean rotation corrections of the *Procrustes* alignment for different camera sets and scale normalization.

Training Data	Test Data				
	HE1 (OP)	H36M (OP)	HE1	H36M	PAN
	rotation error (reduced cam set, no scale norm)				
HE1	23.2°	35.2°	9.3°	31.3°	20.2°
H36M	28.5°	11.2°	22.7°	8.2°	11.0°
PAN	22.3°	18.6°	15.3°	17.1°	4.2°
	rotation error (full cam set, no scale norm)				
HE1	21.3°	35.1°	10.1°	30.6°	13.6°
H36M	26.7°	10.9°	18.1°	7.2°	10.2°
PAN	19.8°	18.6°	12.1°	18.7°	4.1°
	rotation error (full cam set, using scale norm)				
HE1	24.8°	24.3°	8.9°	20.9°	24.1°
H36M	18.1°	12.5°	8.8°	6.5°	9.3°
PAN	18.9°	14.8°	7.7°	8.9°	4.7°

**Table 14:** Error with and without anatomical pose validation, with scale normalization (no-alignment errors in mm, mean ± std. deviation).

Training Data	Test Data				
	HE1 (OP)	H36M (OP)	HE1	H36M	PAN
	no validation				
HE1	138.3 ± 1.3	184.4 ± 4.0	69.2 ± 0.7	170.3 ± 4.0	152.7 ± 2.7
H36M	151.3 ± 1.7	108.6 ± 0.7	86.0 ± 1.2	55.2 ± 0.5	89.2 ± 0.7
PAN	126.1 ± 1.2	130.8 ± 1.1	67.3 ± 1.0	83.1 ± 0.6	37.9 ± 0.4
	using validation				
HE1	125.8 ± 2.4	166.1 ± 4.3	67.5 ± 0.8	155.4 ± 6.6	138.6 ± 2.3
H36M	142.4 ± 1.9	108.3 ± 0.7	84.9 ± 1.3	54.9 ± 0.6	88.9 ± 0.7
PAN	113.9 ± 1.1	130.4 ± 1.0	65.9 ± 1.0	81.8 ± 0.5	37.6 ± 0.4
	mean error change				
HE1	−9.0%	−9.9%	−2.5%	−8.8%	−9.3%
H36M	−5.8%	−0.3%	−1.3%	−0.5%	−0.3%
PAN	−9.7%	−0.3%	−2.1%	−1.6%	−0.7%
	rate of rejected poses				
HE1	15.8%	15.7%	1.8%	13.8%	20.7%
H36M	12.2%	1.3%	1.1%	0.3%	2.2%
PAN	15.6%	2.9%	1.3%	3.3%	0.6%

**Table 15:** No alignment errors of the proposed method compared with Martinez et al. [26]. The proposed method extended Martinez et al. [26] by joint harmonization, scale normalization, some virtual camera augmentation, and, optionally, anatomical pose validation (APV).

Training Data →	HE1			H36M			PAN			
Test Data →	HE1	H36M	PAN	HE1	H36M	PAN	HE1	H36M	PAN	Mean
Martinez et al. [26]	95.9	299.7	148.8	146.0	78.7	107.8	166.7	143.6	43.9	136.8
Proposed	69.2	170.3	152.7	86.0	55.2	89.2	67.3	83.1	37.9	90.1
Proposed + APV	67.5	155.4	138.6	84.9	54.9	88.9	65.9	81.8	37.6	86.2

## Data Availability

The source code for this paper is available at http://added.later (accessed on 27 May 2021). The original baseline model can be found at https://github.com/una-dinosauria/3d-pose-baseline (accessed on 27 May 2021). The Human3.6M dataset can be obtained at vision.imar.ro/human3.6m/ (accessed on 27 May 2021). The Panoptic dataset can be obtained at http://domedb.perception.cs.cmu.edu/ (accessed on 27 May 2021). The Human Eva Dataset can be obtained at http://humaneva.is.tue.mpg.de/ (accessed on 27 May 2021).

## Supplementary Materials

The following are available online at https://www.mdpi.com/article/10.3390/s21113769/s1, Table S1: Errors with original vs. harmonized joints, corresponding to Table 7, Table S2: Errors with reduced vs. full camera set, corresponding to Table 8, Table S3: Error with and without scale normalization, corresponding to Table 9, Table S4: Error of multi-database training with and without scale normalization, corresponding to Table 11.
