# Supplementary material for: A Baseline for Cross-Database 3D Human Pose Estimation
Source: Sensors (Basel). 2021 May 28;21(11):3769. doi: 10.3390/s21113769 (PMC8198914; doi:10.3390/s21113769)
Supplement: Supplementary file 1 [file sensors-21-03769-s001.zip › sensors-1167388-supplementary.pdf]

A Baseline for Cross-Database 3D Human Pose Estimation

Supplemental Material

Michał Rapczyński, Philipp Werner, Sebastian Handrich, and Ayoub Al-Hamadi

Table S1. Errors with original vs harmonized joints (Procrustes errors in mm, mean ± std. deviation) corresponding to Table 7.

Training Data	Test Data HE1	H36M	PAN
	original joints (mean 65.8)		
HE1	59.0 ± 1.1	119.6 ± 3.9	99.0 ± 3.5
H36M	68.2 ± 1.3	47.6 ± 0.3	54.5 ± 1.1
PAN	59.2 ± 0.4	58.2 ± 0.2	27.2 ± 0.3
	harmonized joints (mean 65.1)		
HE1	58.0 ± 1.1	117.9 ± 3.4	98.2 ± 3.5
H36M	67.2 ± 1.3	47.4 ± 0.3	54.1 ± 1.2
PAN	58.3 ± 0.4	57.2 ± 0.2	27.1 ± 0.3
	mean error change		
HE1	-1.7%	-1.4%	-0.8%
H36M	-1.4%	-0.4%	-0.8%
PAN	-1.5%	-1.6%	-0.3%

onesided paired-sample t-test p=0.001

Table S2. Errors with reduced vs full camera set (Procrustes errors in mm, mean ± std. deviation) corresponding to Table 8.

Training Data	Test Data HE1	H36M	PAN
	reduced camera set (mean 75.1)		
HE1	60.5 ± 0.2	136.5 ± 6.5	128.0 ± 3.7
H36M	83.9 ± 8.9	52.1 ± 0.6	66.5 ± 5.2
PAN	59.8 ± 0.4	61.9 ± 0.9	27.0 ± 0.2
	full camera set (mean 65.1)		
HE1	58.0 ± 1.1	117.9 ± 3.4	98.2 ± 3.5
H36M	67.2 ± 1.3	47.4 ± 0.3	54.1 ± 1.2
PAN	58.3 ± 0.4	57.2 ± 0.2	27.1 ± 0.3
	mean error change		
HE1	-4.0%	-13.7%	-23.2%
H36M	-19.9%	-9.1%	-18.6%
PAN	-2.6%	-7.5%	0.6%

onesided paired-sample t-test p=0.008

Table S3. Error with and without scale normalization (no-alignment errors in mm, mean ± std. deviation) corresponding to Table 9.

Training Data	Test Data HE1	H36M	PAN
	no scale normalization (mean 75.1)		
HE1	58.0 ± 1.1	117.9 ± 3.4	98.2 ± 3.5
H36M	67.2 ± 1.3	47.4 ± 0.3	54.1 ± 1.2
PAN	58.3 ± 0.4	57.2 ± 0.2	27.1 ± 0.3
	with scale normalization (mean 60.8)		
HE1	56.8 ± 0.7	103.1 ± 2.2	100.3 ± 2.3
H36M	60.4 ± 0.6	41.3 ± 0.2	48.5 ± 0.9
PAN	54.1 ± 0.8	54.9 ± 0.2	28.1 ± 0.3
	mean error change		
HE1	-2.0%	-12.6%	2.1%
H36M	-10.1%	-12.7%	-10.3%
PAN	-7.2%	-4.1%	3.7%

onesided paired-sample t-test p=0.018

Table S4. Error of multi-database training with and without scale normalization (Procrustes errors in mm, mean ± std. deviation) corresponding to Table 11.

Training Data	Test Data HE1	H36M	PAN
	no scale normalization (mean 55.3)		
H36M + PAN	56.0 ± 0.5	46.9 ± 0.2	27.7 ± 0.2
HE1 + PAN	60.4 ± 0.6	60.2 ± 2.2	29.2 ± 1.2
HE1 + H36M	69.4 ± 2.1	51.8 ± 0.7	57.6 ± 2.0
	with scale normalization (mean 47.9)		
H36M + PAN	50.7 ± 0.3	44.6 ± 0.3	28.6 ± 0.2
HE1 + PAN	54.8 ± 0.4	55.9 ± 0.9	28.7 ± 0.3
HE1 + H36M	66.0 ± 1.2	46.3 ± 1.1	55.8 ± 0.7
HE1	56.8 ± 0.7	103.1 ± 2.2	100.3 ± 2.3
H36M	60.4 ± 0.6	41.3 ± 0.2	48.5 ± 0.9
PAN	54.1 ± 0.8	54.9 ± 0.2	28.1 ± 0.3
	mean error change		
H36M + PAN	-9.4%	-4.9%	3.4%
HE1 + PAN	-9.3%	-7.2%	-1.5%
HE1 + H36M	-4.9%	-10.4%	-3.1%

onesided paired-sample t-test p=0.002
